# The expression of VEGF and cyclin D1/EGFR in common primary liver carcinomas in Egypt: an immunohistochemical study

**DOI:** 10.3332/ecancer.2023.1641

**Published:** 2023-11-27

**Authors:** Dina Sweed, Shaymaa Sabry El Gammal, Shimaa Kilany, Shimaa Abdelsattar, Sara Mohamed Abd Elhamed

**Affiliations:** 1 Pathology Department, National Liver Institute, Shebin Elkom, Menofia University, Shebin Elkom 32511, Menoufia, Egypt; 2 Hepatology and Gastroenterology Department, National Liver Institute, Menoufia University, Shebin Elkom 32511, Menoufia, Egypt; 3 Clinical Biochemistry and Molecular Diagnostics Department, National Liver Institute, Menoufia University, Shebin Elkom 32511, Menoufia, Egypt; a https://orcid.org/0000-0001-6483-5056; b https://orcid.org/0000-0003-0526-2627

**Keywords:** cholangiocarcinoma, cyclin D1, EGFR, hepatocellular carcinoma, VEGF

## Abstract

**Background:**

The most common types of primary malignant liver tumours are hepatocellular carcinoma (HCC) and cholangiocarcinoma (CCA). Treatment options for patients who are inoperable/advanced, or recurring are challenging. Cyclin D1, epidermal growth factor (EGFR) and vascular endothelial growth factor (VEGR) are common carcinogenic proteins that have potential therapeutic targets in various cancers. They have been implicated in the development of HCC and CCA. In this study, we aimed to evaluate the oncogenic function expression of cyclin D1, EGFR and VEGF in HCC and CCA of Egyptian patients. This could help to validate their therapeutic potential.

**Material and methods:**

Tumour cases were selected from 82 cases of primary liver carcinomas, with 58 cases being from HCC and 24 cases from CCA compared to 51 non-tumour adjacent liver cases and 18 from normal liver tissue. The immunohistochemical study of cyclin D1, EGFR and VEGR was conducted.

**Results:**

Cyclin D1, EGFR and VEGF are overexpressed in HCC and CCA as compared to the control group (*p* < 0.001). Cyclin D1 was related to well-differentiated grade and early pathologic stage in HCC (*p* = 0.016 and *p* = 0.042, respectively). The well-differentiated grade showed significantly higher VEGF levels (*p* = 0.04). In the CCA group, however, EGFR was strongly related to high tumour size (*p* = 0.047). EGFR and VEGF were overexpressed in HCC raised in the non-cirrhotic liver compared to those developed in post-hepatitic liver cirrhosis (*p* = 0.003 and *p* = 0.014).

**Conclusion:**

Cyclin D1, EGFR and VEGF shared significant overexpression in HCC and CCA. EGFR and VEGF may play an oncogenic function in the development of HCC in non-cirrhotic liver. Furthermore, cyclin D1 and VEGF may play a good prognostic function in HCC, but EGFR may play a bad prognostic role in CCA.

## Background

Primary liver carcinomas are common malignancies originating from the liver with high mortality and morbidity. Primary liver carcinomas are divided mainly into hepatocellular carcinoma (HCC) represents about 85%, and cholangiocarcinoma (CCA) represents about 10% [1]. Primary liver carcinomas are the sixth most common cancer worldwide, and the fourth leading cause of cancer-related death globally [2]. The highest incidence rates in the world are found in Asia and Africa [3]. In Egypt, HCC is the leading cause of cancer-related mortality among both genders [4]. The major risk factors for HCC vary according to geographic distribution with chronic hepatitis B (HBV) and hepatitis C virus (HCV) infection accounting for 56% and 20% of HCC-related deaths worldwide, respectively. On the other hand, non-viral risk factors including steatotic liver disease a leading cause of HCC in Western countries [5]. CCA risk factors included sclerosing cholangitis, biliary cysts, ulcerative colitis, hepatic lithiasis, hepatic infection and toxins [6]. Despite improved HCV eradication, the incidence of HCC remains elevated due to liver cirrhosis and established genetic abnormalities [7]. CCA cases are increasing annually indicating a need for improved surveillance and management [8].

HCC is potentially curable in its early stages when treated with hepatic resection, transplantation and ablation [5]. The adoption of treatment stage migration (TSM) was proposed in the 2022 update of The Barcelona Clinic Liver Cancer system treatment algorithm of HCC. TSM is used when a certain patient profile or therapy failure/infeasibility may cause the advice to shift to the alternative that would be considered for a more advanced stage [9]. Targeted agents are frequently associated with significant resistance and adverse effects. Furthermore, for advanced patients, chemotherapy lacks its survival advantage [10]. Sorafenib is well-established as a targeted therapy for late-stage HCC; however, it is complemented by a high risk of tumour resistance [11].

Radical surgery with a negative resection margin is the best curative management for CCA with liver transplantation not considered a standard treatment. Local regional therapy such as ablation and transarterial chemoembolisation could be optional therapies in advanced liver-limited disease. However, 40%–85% of patients experience recurrence of the disease following radical excision [12]. In addition, CCA has a very poor prognosis with no current effective pharmacological treatment available [13].

HCC and CCA share some commonly known risk factors and possible carcinogenic pathways [14]. Cyclin D1 is a cyclin-dependent kinase (CDK) 4/6 regulatory protein. Cyclin D1 increases cell proliferation, and its activation is assumed to be the first step in the progression of HCC and CCA through enhanced cell-cycle progression [13, 15]. Receptor tyrosine kinases (RTKs) are being recognised as important participants in tumour progression and cancer dissemination [16]. Some RTKs, such as epidermal growth factor (EGFR), can contribute to tumour spreading by establishing a molecular complex with integrin, inducing genetic aberrations and inducing treatment resistance [17]. Targeting EGFR may participate in the treatment of primary liver cancer [18].

Angiogenesis is an essential step in the development of HCC. Vascular endothelial growth factor (VEGF) is regarded as a driving factor in both healthy and pathological angiogenesis [19]). Sorafenib, a VEGF inhibitor, has been found to enhance survival in late-stage HCC. Resistance to anti-VEGF therapy, on the other hand, prompts research into combinational or alternative medications [20]. Although biliary tract inflammation is the first step in carcinogenesis, the microenvironment also plays an important role in pathogenesis, encouraging tumour angiogenesis and spread. VEGF plays a role in angiogenesis and has been studied as a prognostic marker in CCA [21].

As a result, we designed this study to study the protein expression of cyclin D1, EGFR and VEGF in Egyptian patients with HCC and CCA with the prognostic factors. Furthermore, determining the expression level of these proteins could help to elucidate their therapeutic potential.

## Material and methods

This is retrospective, case-control research conducted at the Pathology Department, National Liver Institute, Egypt. The cases were obtained as part of the patients' medical management between December 2020 and December 2022. Tumour samples were collected from 82 primary liver cancer patients who were candidates for curative surgical resection. The cases were divided into 58 HCC cases and 24 CCA cases. As a control, 51 cases of adjacent non-tumour liver tissue and 18 cases of healthy liver tissue were included. The control, healthy group included potential liver transplant donors with normal liver function tests and ultrasound findings. Serological results for autoimmune and viral liver disorders were negative, and there was no history of diabetes or metabolic syndrome. Upon institution approval (NLIIRB protocol Number 00485/2023), clinical and survival information was obtained.

After surgery, there will be a 1-month follow-up with ultrasound, triphasic computed tomography and serum tumour markers, followed by a 2-month follow-up and then 3-month follow-ups for 1 year. Then every 6 months for 3 years. The overall survival (OS) was calculated from the diagnosis date through the last follow-up visit or death.

## Inclusion criteria

Primary liver carcinomas approved to be classic HCC or CCA based on clinical, laboratory, pathological and immunohistochemical confirmation.

## Exclusion criteria

Primary liver carcinomas in children are not considered. All patients who had undergone local ablation or had systemic therapy such as neo-adjuvant chemotherapy or sorafenib before surgery were excluded.

### Pathological studies

Tumour size and multiplicity, pathological grade, lymph vascular invasion (LVI), perineural invasion and pathological stage were all included in the pathological data for tumour cases. Using the tumour-node-metastasis classification method, the pathologic stage was evaluated. The following was the pathological stage for HCC: T1a, solitary ≤ 2 cm on greatest dimension with or without LVI, T1b, solitary > 2 cm on greatest dimension without LVI, T2, solitary > 2 cm on greatest dimension with LVI or multiple tumours, none > 5 cm on greatest dimension, T3, multiple tumours any > 5 cm on greatest dimension, T4, tumour(s) involving a major branch of the portal or hepatic vein or with direct invasion of adjacent organs or with perforation of visceral peritoneum. The following was the pathological stage for CCA: T1a, solitary ≤ 5 cm on greatest dimension without LVI, T1b, solitary > 5 cm on greatest dimension without LVI, T2, solitary tumour with LVI or multiple tumours with or without LVI, T3, tumour perforating the visceral peritoneum, T4, tumour involving local extrahepatic structures by direct hepatic invasion [22, 23].

### Immunohistochemical technique

The tissue microarray technique was done for both tumour and non-tumour tissue samples [24]. For the normal liver tissue, sections from needle liver biopsy samples were used.

The cyclin D1, EGFR and VEGF primary antibodies were all acquired from (Bioss, Massachusetts, USA) and diluted at 1:200. Sections were treated for 20 minutes in high PH Dako antigen retrieval (Ref K8000) before cooling for another 20 minutes. The slides were covered by primary antibodies overnight at 4°C. The immunostaining was visualised using 3-diaminobenzidine chromogen (DAKO A/S). For each run, positive and negative controls were used. Cyclin D1 revealed nuclear expression while EGFR and VEGF revealed cytoplasmic expression. The histoscore (*H* score) approach has been used by two blinded histopathologists to independently evaluate the expression of markers. *H* score is calculated via multiplication of a percentage by the intensity (0–3): *H*-score = [(0 × % negative cells) + (1 × % mildly positive cells) + (2 × % moderately positive cells) + (3 × % strongly positive cells)] with a total score of 0 to 300 [25].

### Statistics

The Statistical Package for the Social Sciences software version 20.0 was used to analyse the data. The chi-square test, along with Fisher exact and Monte Carlo correction tests, was used to compare the two groups. The normality of continuous data was investigated. For abnormally distributed quantitative variables, the Mann-Whitney and Kruskal Wallis tests were used to compare two groups and more than two studied groups, respectively. For paired comparisons, use *post hoc* (Dunn's multiple comparisons test). The Pearson correlation coefficient was applied to examine the relationship between the two variables. The multivariate Cox proportional hazard regression model includes factors that were significantly associated with OS. The significance of the results was assessed at the 5% level.

## Results

Table 1 shows the detailed clinicopathological data for both tumour groups.

### The expression of cyclin D1, EGFR and VEGF in the tumour and control groups

In the normal control group, cyclin D1 was observed in 40% of cases, while EGFR and VEGF were detected in 88.9% and 61.1%, respectively. Cyclin D1 was observed in 40% of cases in adjacent non-tumour liver, while EGFR and VEGF were detected in 88.9% and 61.1%, respectively.

In HCC cases, cyclin D1 was observed in 84.3% of cases, while EGFR and VEGF were detected in 96.1% and 66.7%, respectively.

In CCA cases, cyclin D1 was observed in 95.8% of cases, while EGFR and VEGF were detected in 100% and 91.7%, respectively. Detailed markers expression is illustrated in Table 2.

**Figure 1.**
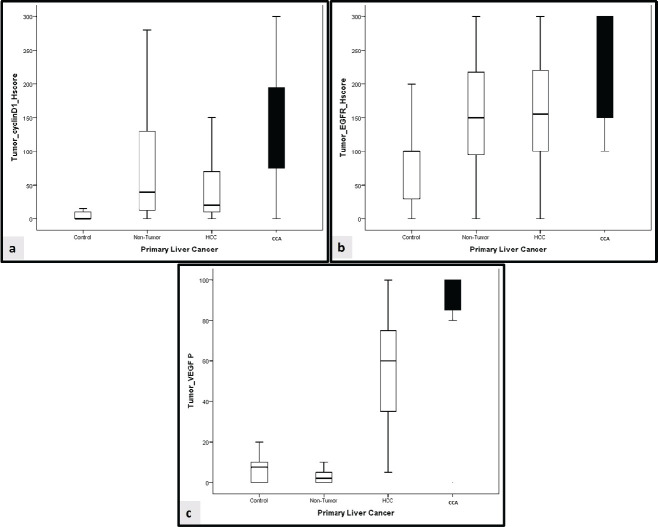
Comparison between cyclin D1, EGFR and VEGF in the studied groups. (a): Comparative expression of cyclin D1 H score between the studied groups. (b): Comparative expression of EGFR H score between the studied groups. (c): Comparative expression of VEGF H score between the studied groups. HCC: Hepatocellular carcinoma, CCA: cholangiocarcinoma.

**Figure 2.**
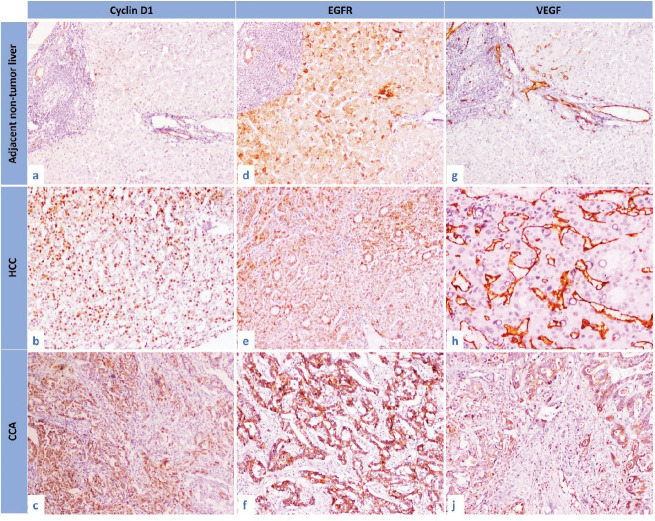
The immunohistochemical expression of cyclin D1, EGFR and VEGF in the studied groups. (a): Cyclin D1 expression in adjacent non-tumour liver, (b): cyclin D1 expression in HCC, (c): cyclin D1 expression in CCA, (d): EGFR expression in adjacent non-tumour liver, (e): EGFR expression in HCC, (f): EGFR expression in CCA, (g): VEGF expression in adjacent non-tumour liver, (h): VEGF expression in HCC and (i): VEGF expression in CCA (IHC, 200×, for all).

In comparison to the control group, cyclin D1 *H* score was overexpressed in non-tumorous liver tissue and tumour groups (*p* < 0.001, for both). Similarly, overexpression of EGFR *H* score was significantly observed in non-tumorous liver tissue and tumour groups (*p* = 0.007 and *p* < 0.001, respectively). Furthermore, the VEGF *H* score was significantly overexpressed in the tumour group (*p* < 0.001, for both) (Figures 1 and 2).

### Correlation between the cyclin D1, EGFR and VEGF in the primary liver carcinoma groups

**Figure 3.**
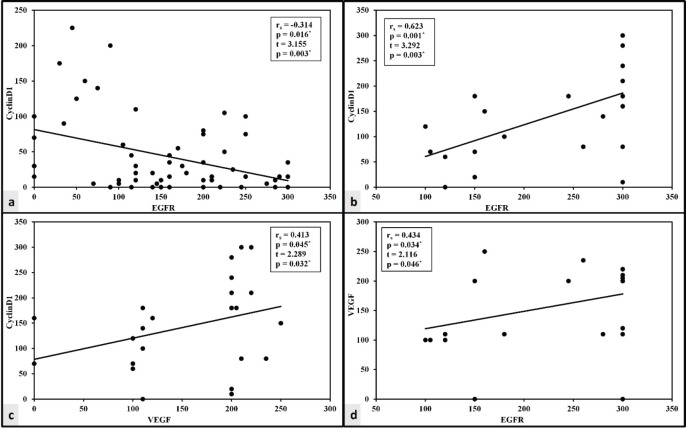
Correlation between the studied markers in HCC and CCA groups. (a): Correlation between cyclin D1 and EGFR in HCC group. (b): Correlation between cyclin D1 and EGFR in CCA group. (c): Correlation between cyclin D1 and VEGF in CCA group. (d): Correlation between EGFR and VEGF in CCA group.

There was a lack of correlation between VEGF and either cyclin D1 or EGFR in the HCC group. Cyclin D1 and the EGFR *H* score of expression, however, have a negative correlation. The expression of three markers exhibited a significant positive correlation in the CCA group (Figure 3).

This is confirmed using the linear regression analysis (Table 3).

### Relation between the studied markers and the different clinicopathological parameters in tumour groups

The female gender and old age were both significantly linked with the cyclin D1 *H* score in the HCC group (*p* = 0.032 and *p* = 0.011, respectively). A high cyclin D1 *H* score was also strongly related to a well-differentiated grade and an early tumour stage (*p* = 0.016 and *p* = 0.042, respectively VEGF *H* score was significantly higher in well-differentiated grade (*p* = 0.040) (Table 4).

The EGFR *H* score had a significant association with large tumour size in the CCA group (*p* = 0.047), as shown in Table 5.

### Multivariate COX regression analysis for the parameters affecting mortality in primary liver carcinomas

Furthermore, multivariate COX regression analysis for factors influencing cancer mortality did not reveal any effect of clinicopathological or marker expression on patient survival in cases of primary liver carcinoma, as shown in Table 6.

### Regarding the etiological background of the adjacent non-tumour liver

There was significant overexpression of EGFR and VEGF in HCC raised in the non-cirrhotic (virology negative) liver compared to those developed in post-hepatitic liver cirrhosis (*p* = 0.003 and *p* = 0.014) (Table 3).

## Discussion

In the current study, HCC and CCA shared overexpression of cyclin D1, EGFR and VEGF with the dominant expression in the CCA group. Furthermore, there is a lack of significant correlation between cyclin D1/EGFR and VEGF expression in HCC cases, in contrast to their positive correlation with VEGF expression in CCA.

Through the activation of cell cycle progression, the expression of cyclin D1 in the HCC and CCA groups in the current study may enhance cell proliferation. Cyclin D1 is created in the G1 phase, where it then interacts with CDK4/6 to control the G1/S phase transition. In HCC, cyclin D1 overexpression could be induced through gene amplification [26, 27]. Other studies emphasise the regulatory role of the autophagy pathway on cyclin D1 activation [26, 28]. Similarly, CCA overexpressed cyclin D1 to evade the inhibitory effect of transformed growth factor-β [29]. Another *in-vitro* study found that cyclin D1-enriched CCA tissue significantly reduced the count of cells that were exposed to CDK4/6 inhibitors [13].

The EGFR has also been linked to the development of HCC and CCA tumours in previous studies [30, 31]. EGFR overexpression is common in HCC, and its activation could be an alarming sign of primary resistance to sorafenib [32]. EGFR family members were expressed in CCA contributing to tumour development and aggressiveness [33]. In addition, angiogenesis is considered a cornerstone in the progression and metastasis of human HCC [34]. Angiogenesis occurs when tumour microenvironmental cells, hepatic stellate cells and inflammatory cells begin to release VEG [35]. VEGF has been reported to be overexpressed in different classes of CCA (intrahepatic and extrahepatic) [36]. We postulate that the treatment of HCC and CCA malignancies may be affected by the overexpression of cyclin D1 and EGFR.

In the CCA group, the present study showed a direct association between cyclin D1/EGFR and VEGF. The production of cyclin D1 induces the activation of VEGF, which then modulates blood vessels. Cyclin D1 modulates STAT 3 activity and so promotes VEGF production [37]. Cyclin D1 and VEGF are the downstream target proteins of the NF-κB pathway in CCA cells [38]. Additionally, the VEGF and EGFR pathways both share downstream carcinogenic signalling [39]. VEGF signalling is upregulated by EGFR expression [40].

In HCC and CCA cases, there was a controversial relationship between cyclin D1 and EGFR. This conflict was observed in previous studies. Hernandez-Garcia *et al* [41] found EGFR tyrosine-phosphorylation and activation in the cyclin D1 expressed HCC cell lines and HCC samples. The possible mechanism could be through activating the EGFR/Akt/NF-κB/cyclin D1 [42, 43]. A negative association between EGFR and cyclin D1 in HCC, on the contrary, could be the result of alternative pathway activity. EGFR activation leads to β-catenin phosphorylation mediated by PI3K leading to decreased expression of cyclin D1 [44].

HCC raised in the non-cirrhotic liver significantly overexpressed EGFR and VEGF in comparison to those developed in post-hepatitic liver cirrhosis. There is debate regarding the functional relevance of VEGF in HCC amongst patient groups with and without cirrhosis. According to Fodor *et al* [45], VEGF plays a detrimental function in the development of immature vasculature in individuals with cirrhosis. However, other studies found high serum VEGF expression in HCC on top of the non-cirrhotic liver and assumed that the downregulation of VEGF levels in patients with portal hypertension impacts hepatocyte regeneration [46]. There is little information available regarding the relative expression of EGFR and cyclin D1 on HCC that develops in livers with and without cirrhosis. The involvement of EGFR in HCC could be altered by EGFR overexpression or functional polymorphism with no accompanying copy number gain [47].

The potential therapeutic role of VEGF, cyclin D1 and EGFR has been reported in association with sorafenib therapy. Sorafenib prevents tumour cell growth and angiogenesis, which significantly slows the course of HCC and increases patient survival time [48]. Sorafenib's antiangiogenic effect could be mediated through VEGFR activation [49, 50]. In addition, sorafenib reduces the production of cyclin D1 and arrests the cell cycle to prevent the proliferation of tumour cells [11, 51]. On the contrary, EGFR activation could induce sorafenib resistance in primary HCC cells [32]. As a result, decreasing EGFR expression or limiting its kinase activity could increase sorafenib sensitivity [52].

Regarding the prognostic role of the studied markers, cyclin D1 and VEGF shared a similar good prognostic impact on the HCC group which agrees with the previous studies. It may be possible to determine an early role for cyclin D1 in hepatocarcinogenesis and tumour differentiation from the significant association between cyclin D1 expression and a well-differentiated HCC histology [53]. Well-differentiated HCCs had the highest levels of VEGF expression, which was followed by moderately and poorly differentiated HCCs [54, 55]. On the other hand, other studies found an association between cyclin D1 and high-grade and advanced stages of HCC [15, 56]. In CCA, EGFR was associated with large tumour size which could be explained by the role of EGFR in induction of the proliferative activity and tumour growth [57, 58]. Cyclin D1 and VEGF did not significantly correlate with the prognostic factors in CCA, which may be due to the small sample size of the studied cases. In CCA, VEGF expression is linked to poor prognostic indicators such as nodal metastases and advanced stage [59].

## Conclusion

Overexpression of cyclin D1, EGFR and VEGF in HCC and CC contributes to their pathogenesis. Cyclin D1, EGFR and VEGF shared significant overexpression in HCC and CCA. EGFR and VEGF may play an oncogenic function in the development of HCC in non-cirrhotic liver. Furthermore, Cyclin D1 and VEGF may play a good prognostic function in HCC, but EGFR may play a bad prognostic role in CCA.

## Limitations and future recommendations

Future genetic studies are recommended to determine the wild/mutant form of EGFR in relation to VEGF. Clinical investigations are recommended to determine the value of sorafenib in combination with cyclin D1 and/or EGFR inhibitors in the management of sorafenib-resistant HCC and CCA.

## Conflicts of interest

The authors declare no conflicts of interest.

## Funding

This study received no specific funding from government, commercial, or non-profit organisations.

## Ethics approval

The study was approved by the ethics committee/Institutional Review Board of (National Liver Institute, Menoufia University, (NLIIRB protocol Number 00485/2023)).

## Author contributions

DS, wrote the manuscript, contributed to the study design and supervised the implementation of the research and corresponding author; SSE, implemented the research and contributed to writing the manuscript; SK, contributed to the concept and design of the work, performed the clinical part and collecting the data and agreed on the final version; SA, contributed to the concept and design of the work, and revising the paper; SMA, contributed to study design, supervised implementing the research, and revising writing process of the manuscript.

## Figures and Tables

**Table 1.** Comparison between the HCC and CCA according to different parameters.

	HCC (*n* = 58)	CCA (*n* = 24)	Test of sig.	*p*
Age				
Mean ± SD	57.3 ± 5.66	53.6 ± 11.3	*t* = 1.529	0.138
Median (min–max)	58 (42–72)	56 (34–73)		
Sex				
Male	46 (79.3%)	15 (62.5%)	*χ*^2^ = 2.518	0.113
Female	12 (20.7%)	9 (37.5%)		
AFP				
<200	43 (76.8%)	8 (88.9%)	*χ*^2^ = 0.672	*p* = 0.670^FE^
>200	13 (23.2%)	1 (11.1%)		
Aetiology HCV infection Non-HCV, non-HBV	55 (94.8%) 3 (5.2%)	8 (33.3%) 16 (66.7%)	*χ*^2^ = 36.060	<0.001
Focality				
Solitary	44 (75.9%)	20 (83.3%)	*χ*^2^ = 0.553	0.457
Multiple	14 (24.1%)	4 (16.7%)		
Tumour size				
<5 cm	9 (15.5%)	11 (45.8%)	*χ*^2^ = 8.460^*^	0.004^*^
>5 cm	49 (84.5%)	13 (54.2%)		
Grade				
1	50 (86.2%)	9 (37.5%)	*χ*^2^ = 19.555^*^	<0.001^*^
2	8 (13.8%)	15 (62.5%)		
Background liver Non-cirrhosis	19 (32.8%)	19 (79.2%)	*χ*^2^ = 14.703^*^	<0.001^*^
Cirrhosis	39 (67.2%)	5 (20.8%)		
Stage				
Early	50 (86.2%)	21 (87.5%)	*χ*^2^ = 0.024	*p* = 1.000^FE^
Late	8 (13.8%)	3 (12.5%)		
LVI				
Negative	30 (57.1%)	15 (62.5%)	*χ*^2^ = 0.796	0.372
Positive	28 (48.3%)	9 (37.5%)		
Perineural invasion				
Negative	58 (100%)	15 (62.5%)	*χ*^2^ = 24.432^*^	*p* < 0.001^*^^FE^
Positive	0 (0%)	9 (37.5%)		

SD: Standard deviation, HCC: hepatocellular carcinoma, CCA: cholangiocarcinoma, AFP: alpha-fetoprotein, HCV: hepatitis C virus, HBV: hepatitis B virus, LVI: lymph vascular invasion, *χ*^2^: Chi-square test, MC: Monte Carlo, FE: Fisher exact, *U*: Mann Whitney test, *p*: *p*-value for comparing between the studied groups

* Statistically significant at *p* ≤ 0.05

**Table 2.** Comparison between the three studied groups according to different markers.

	Normal (*n* = 18)	Non-tumour (*n* = 51)	Primary liver carcinomas (*n* = 82)	Test of sig.	*p*	Sig. bet. grps.	OR (LL–UL 95% CI)
Cyclin D1							
Negative	11 (61.1%)	8 (15.7%)	11 (13.4%)	*χ*^2^ = 21.937	<0.001^*^	*p*_1_ = 0.716 *p*_2_ < 0.001^*^^FE^ *p*_3_ = 0.716	10.143^*^(3.241–31.738)
Positive	7 (38.9%)	43 (84.3%)	71 (86.6%)				
Cyclin D1 *H* score							
Mean ± SD	11.7 ± 22.4	75.3 ± 82.4	73.8 ± 79.2	*H* = 18.499^*^	<0.001^*^	*p*_1_ < 0.001^*^ p_2_ < 0.001^*^ p_3_ = 0.984	1.035^*^(1.008–1.063)
Median (min–max)	0 (0–80)	40 (0–280)	40 (0–300)				
EGFR							
Negative	2 (11.1%)	2 (3.9%)	5 (6.1%)	*χ*^2^ = 1.491	*p* = 0.470^MC^	*p*_1_ > 0.050 *p*_2_ > 0.050 *p*_3_ > 0.050	1.925 (0.343–10.815)
Positive	16 (88.9%)	49 (96.1%)	77 (93.9%)				
EGFR *H* score							
Mean ± SD	94.4 ± 76.4	156.3 ± 95.9	177.4 ± 91.6	*H* = 13.069^*^	0.001^*^	*p*_1_ = 0.007^*^ p_2_ < 0.001^*^ p_3_ = 0.248	1.011^*^(1.004–1.018)
Median (min–max)	100 (0–300)	150 (0–300)	165 (0–300)				
VEGF							
Negative	7 (38.9%)	17 (33.3%)	2 (2.4%)	*χ*^2^ = 27.788^*^	<0.001^*^	*p*_1_ < 0.001^*^ p_2_ < 0.001^*^^FE^ *p*_3_ = 0.670	25.455^*^(4.682–138.385)
Positive	11 (61.1%)	34 (66.7%)	80 (97.6%)				
VEGF *H* score							
Mean ± SD	24.2 ± 26.3	7.8 ± 8.1	164.8 ± 71.5	*H* = 101.059^*^	<0.001^*^	*p*_1_ = 0.322 *p*_2_ < 0.001^*^ p_3_ < 0.001^*^	1.045^*^(1.023–1.068)
Median (min–max)	22.5 (0–90)	6 (0–30)	187.5 (0–300)				

SD: Standard deviation, *χ*^2^: chi-square test, MC: Monte Carlo, FE: Fisher exact, EGFR: epidermal growth factor, receptor, VEGF: vascular endothelial growth factor, OR: odds ratio, CI: confidence interval, LL: lower limit, UL: upper limit, *H*: *H* for Kruskal Wallis test, pairwise comparison between each two groups was done using a *post hoc* test (Dunn's for multiple comparisons test), *p*: *p*-value for comparing between the studied groups

*p*_1_: *p*-value for comparing between normal and non-tumour

*p*_2_: *p*-value for comparing between normal and primary liver cancer

*p*_3_: *p*-value for comparing between non-tumour and primary liver cancer

* Statistically significant at *p* ≤ 0.05

**Table 3.** Linear regression analysis of the relationship between cyclin D1, EGFR and VEGF in primary liver carcinomas.

	Group	B	SE	Beta	*t*	*p*	*R* ^2^	Adjusted *R*^2^	*F*	*p*
EGFR versus cyclin D1	HCC	−0.627	0.199	−0.388	3.155	0.003^*^	0.151	0.136	9.952^*^	0.003^*^
EGFR versus cyclin D1	CCA	0.526	0.160	0.574	3.292	0.003^*^	0.330	0.300	10.837^*^	0.003^*^
VEGF versus cyclin D1	CCA	0.353	0.154	0.439	2.289	0.032^*^	0.192	0.156	5.240	0.032^*^
EGFR versus VEGF	CCA	0.362	0.171	0.411	2.116	0.046^*^	0.169	0.131	4.476	0.046^*^

HCC: Hepatocellular carcinoma, CCA: cholangiocarcinoma, *F*, *p*: *f* and *p* values for the model, *R*^2^: coefficient of determination, *R*: coefficient of regression, *B*: unstandardised coefficients, SE: estimates standard error, Beta: standardised coefficients, *t*: *t*-test of significance

**Table 4.** Relation between cyclin D1, EGFR and VEGF expression in the HCC group.

Variables	Cyclin D1		EGFR		VEGF	
	Mean ± SD	Median	Mean ± SD	Median	Mean ± SD	Median
Sex		(min–max)		(min–max)		(min–max)
Male	39.2 ± 50.9	15 (0–225)	147.7 ± 87.5	147.5 (0–300)	161.1 ± 74.5	180 (15–270)
Female	64.2 ± 60.9	45 (10–200)	177.5 ± 81.2	187.5 (30–300)	191.3 ± 61.4	187.5 (120–300)
*U* (*p*)	164.50^*^ (0.032^*^)		224.0 (0.318)		224.0 (0.316)	
AFP						
<200	39.7 ± 49.7	20 (0–200)	169.4 ± 81.3	160 (0–300)	172.7 ± 71.1	195 (30–285)
>200	61.5 ± 65.7	30 (0–225)	113.9 ± 88	140 (0–250)	160.4 ± 72	180 (75–300)
*U* (*p*)	215.0 (0.209)		185.50		233.50 (0.370)	
Focality						
Solitary	50.2 ± 56.2	30 (0–225)	159 ± 84.6	160 (0–300)	166.7 ± 73.9	180 (15–300)
Multiple	26.1 ± 40.2	12.5 (0–150)	137.9 ± 93.3	135 (0–300)	169.3 ± 70.5	195 (45–240)
*U* (*p*)	216.50 (0.095)		263.0 (0.413)		295.50 (0.820)	
Tumour size						
<5 cm	51.1 ± 45.4	35 (10–150)	189.4 ± 54.5	200 (60–250)	200 ± 60.5	225 (90–270)
>5 cm	43.2 ± 55.2	20 (0–225)	147.4 ± 89.9	140 (0–300)	161.3 ± 73.5	180 (15–300)
*U* (*p*)	165.0 (0.231)		149.50 (0.127)		142.0 (0.090)	
Grade						
1	44.6 ± 51.8	22.5 (0–225)	164.6 ± 80	160 (0–300)	174.3 ± 72.2	195 (15–300)
2	43.1 ± 67.1	15 (0–200)	86.9 ± 100.2	80 (0–300)	123.8 ± 62	127.5 (45–195)
*U* (*p*)	95.0^*^ (0.016^*^)		181.0 (0.682)		109.50^*^ (0.040^*^)	
Non-cirrhosis	32.1 ± 39.5	15 (0–140)	202.6 ± 86.8	210 (0–300)	201 ± 1	225 (90–285)
Cirrhosis	50.4 ± 58.7	30 (0–225)	130.1 ± 76.5	120 (0–285)	150.4 ± 73.7	150 (15–300)
*U* (*p*)	297.50 (0.225)		191.0^*^ (0.003^*^)		222.50^*^ (0.014^*^)	
Stage						
Early	45.6 ± 54.3	22.5 (0–225)	162.8 ± 82.4	160 (0–300)	172.8 ± 71.5	187.5 (15–300)
Late	36.9 ± 50.7	15 (0–150)	98.1 ± 95.2	92.5 (0–300)	133.1 ± 74.1	127.5 (45–225)
*U* (*p*)	110.50^*^ (0.042^*^)		179.0 (0.650)		135.0 (0.148)	
LVI						
Negative	40.7 ± 60.3	15 (0–225)	159.3 ± 80	160 (0–300)	156.5 ± 72.6	165 (15–270)
Positive	48.4 ± 45.9	32.5 (0–150)	148 ± 93.9	135 (0–300)	178.9 ± 71.9	187.5 (30–300)
*U* (*p*)	388.50 (0.624)		335.50 (0.187)		352.50 (0.292)	
Perineural invasion						
Negative	44.4 ± 53.5	20 (0–225)	153.9 ± 86.4	155 (0–300)	167.3 ± 72.5	180 (15–300)
Positive	–	–	–	–	–	–
*U* (*p*)	–		–		–	

SD: Standard deviation, *U*: Mann Whitney test, *H*: *H* for Kruskal Wallis test, *p*: *p*-value, EGFR: epidermal growth factor receptor, VEGF: vascular endothelial growth factor, HCC: hepatocellular carcinoma, AFP: alpha-fetoprotein, LVI: lymph vascular invasion

* Statistically significant at *p* ≤ 0.05

**Table 5.** Relation between cyclin D1, EGFR and VEGF expression in the CCA group.

Variables	Cyclin D1		EGFR		VEGF	
	Mean ± SD	Median	Mean ± SD	Median	Mean ± SD	Median
		(min–max)		(min–max)		(min–max)
Sex						
Male	151.3 ± 94.9	150 (0–300)	237.7 ± 78.2	280 (100–300)	158.3 ± 71.2	200 (0–250)
Female	134.4 ± 75.9	160 (20–240)	228.3 ± 86.1	300 (105–300)	159.4 ± 72.2	200 (0–210)
*U* (*p*)	63.500 (0.815)		67.500 (1.000)		64.0 (0.861)	
Focality						
Solitary	132 ± 84.9	135 (0–300)	222 ± 81.8	252.5 (100–300)	158 ± 73.8	200 (0–250)
Multiple	210 ± 73.9	200 (140–300)	295 ± 10	300 (280–300)	162.5 ± 55.6	160 (110–220)
*U* (*p*)	21.0 (0.157)		22.500 (0.183)		37.0 (0.852)	
Tumour size						
<5 cm	138.2 ± 93.9	140 (0–300)	195 ± 82.8	160 (100–300)	144.6 ± 72.9	110 (0–250)
>5 cm	150.8 ± 83.9	180 (10–300)	267.3 ± 61.7	300 (120–300)	170.8 ± 68	200 (0–235)
*U* (*p*)	61.0 (0.569)		37.0^*^ (0.047^*^)		51.500 (0.252)	
Grade						
1	126.7 ± 85.3	100 (20–300)	212.8 ± 85.5	180 (105–300)	167.2 ± 60.8	200 (100–250)
2	156 ± 88.9	160 (0–300)	247 ± 75.7	300 (100–300)	153.7 ± 76.6	200 (0–235)
*U* (*p*)	57.500 (0.558)		52.500 (0.379)		60.0 (0.682)	
Non-cirrhosis	139.5 ± 89.5	150 (0–30)	223.2 ± 82.6	260 (100–300)	162.6 ± 66.2	200 (0–250)
Cirrhosis	166 ± 81.7	160 (80–280)	276 ± 53.7	300 (180–300)	144 ± 90.2	200 (0–210)
*U* (*p*)	38.50 (0.534)		27.0 (0.160)		41.50 (0.679)	
Stage						
Early	136.7 ± 89.1	140 (0–300)	224.8 ± 80.8	260 (100–300)	155.7 ± 72.7	200 (0–250)
Late	203.3 ± 40.4	210 (160–240)	300 ± 0	300 (300–300)	180 ± 52.9	200 (120–220)
*U* (*p*)	13.500 (0.122)		15.0 (0.172)		23.500 (0.505)	
LVI						
Negative	122.7 ± 84.8	100 (10–300)	210.3 ± 81.7	180 (100–300)	162.7 ± 70.5	200 (0–250)
Positive	182.2 ± 81.4	180 (0–300)	273.9 ± 60.5	300 (120–300)	152.2 ± 72.9	200 (0–220)
*U* (*p*)	35.50 (0.055)		37.0 (0.073)		64.50 (0.861)	
Perineural invasion						
Negative	150.7 ± 88.6	160 (10–300)	253.7 ± 69.8	300 (120–300)	161.7 ± 79.1	200 (0–250)
Positive	135.6 ± 88.3	120 (0–300)	201.7 ± 88	180 (100–300)	153.9 ± 55.7	120 (100–235)
*U* (*p*)	43.0 (0.155)		61.0 (0.726)		58.500 (0.599)	

SD: Standard deviation, *U*: Mann Whitney test, *H*: *H* for Kruskal Wallis test, *p*: *p*-value, EGFR: epidermal growth factor receptor, VEGF: vascular endothelial growth factor, CCA: cholangiocarcinoma, LVI: lymph vascular invasion

* Statistically significant at *p* ≤ 0.05

**Table 6.** Multivariate COX regression analysis for the parameters affecting mortality in primary liver carcinomas.

	*p*	HR (LL–UL 95% CI)
Focality (multiple)	0.117	0.080 (0.003–1.883)
Tumour size (>5)	0.633	1.461 (0.308–6.928)
Grade	0.669	1.580 (0.194–12.864)
Stage (late)	0.120	17.242 (0.476–624.061)
LVI	0.756	0.837 (0.273–2.571)
Cyclin D1 *H* score	0.784	0.998 (0.986–1.011)
EGFR *H* score	0.243	1.004 (0.997–1.012)
VEGF *H* score	0.192	1.005 (0.997–1.013)

HR: Hazard ratio, CI: confidence interval, LL: lower limit, UL: upper, EGFR: epidermal growth factor receptor, VEGF: vascular endothelial growth factor, LVI: lymph vascular invasion
